# A systematic review on the metaverse-based blended English learning

**DOI:** 10.3389/fpsyg.2022.1087508

**Published:** 2023-01-06

**Authors:** Ming Li, Zhonggen Yu

**Affiliations:** Faculty of Foreign Studies, Beijing Language and Culture University, Beijing, China

**Keywords:** metaverse, blended English learning, learner engagement, learning outcome, digital literacy, challenges

## Abstract

Over the past two decades, various digital technologies have been applied to sustain higher education. As the latest emerging information technology, the metaverse has been a recurring theme to be considered as a new direction to promote blended English learning. This study aims to investigate metaverse-based blended English learning. Through a systematic review based on bibliographic and content analysis, the study attempts to integrate the evidence to generate a model that links the education-based metaverse. The metaverse platforms in which learners' academic success can be significantly enhanced due to a high degree of learner engagement in immersive virtual environments. In addition, the virtual learning experience is restricted by the degree of digital literacy at the same time. To improve instructors' and learners' digital literacy levels, necessary support is indispensable by educational institutions and designers of the metaverse platforms. Meanwhile, this study addresses potential challenges that may hinder sustaining metaverse-based blended English learning, and provides some suggestions based on the previous literature. In future research, we will keep updating and polishing the metaverse-based blended English learning research to provide more detailed guidance for researchers and educators.

## Introduction

Over the past two decades, various digital technologies have been applied to sustain higher education (Kye et al., 2021). The significance of technological advancements has been highlighted to support digital learning during the post-pandemic era (Mughal et al., 2022). Recently, the metaverse has been a recurring theme to be considered a new direction to promote education (Jovanović and Milosavljević, 2022). The metaverse as a three-dimensional-based (3D) virtual reality has been considered to adapt to education, especially blended learning, although the metaverse is not a new concept (Dayoub, 2022). Researchers and educators have discussed the implication of the metaverse for blended learning (Kye et al., 2021). The COVID-19 pandemic accelerated the acceptance of the metaverse technology in some leading universities in China and worldwide (Chong et al., 2014; Devlin and McKay, 2016; Verma and Dahiya, 2016; Reisoglu et al., 2017; Pastor et al., 2020; Vershitskaya et al., 2020; Li et al., 2022; Mughal et al., 2022). It is necessary to investigate the effects of the metaverse on blended learning.

Moreover, metaverse-based technology plays a significant role in blended learning in higher education contexts (Díaz et al., 2020). It allows learners to interact with each other socially (Puncreobutr et al., 2022). The metaverse-based technology facilitates learning regardless of learners' location, tacitly promoting learners' academic performance, coupled with leisure and education (Li and Yu, 2022). A different aspect of the metaverse compared to traditional information and communication technology is that it creates a virtual world outside the traditional classroom (Puncreobutr et al., 2022). The immersive virtual world can potentially improve active and creative learning, as learners have been no strangers to the virtual environment since the beginning of 2020 (Puncreobutr et al., 2022). The visual aspect of the metaverse and the possibility of real-time change benefit learning by amplifying learner imagination and intelligence (Ryu et al., 2022). Therefore, there is a need to explore integrating metaverse-based technology into learning.

However, the metaverse has not been fully explored (Davis et al., 2009). The metaverse is still in its infancies (Reisoglu et al., 2017). A lack of literature summarized the findings of immerging the metaverse with blended English learning and even less provided essential insights into current and future English instructional guidance (Tlili et al., 2022a,b). Hence, several questions have not been answered, such as what assessing factors are used in the metaverse; or what challenges are in adopting the metaverse to blended English learning. Consequently, this study aims to conduct a systematic review of the metaverse applied to blended English learning to fill the research gap. This study conducts bibliometric and content analysis to investigate metaverse-based blended English learning.

This study aims to investigate metaverse-based blended English learning and provide guidance for current English education in higher education. As blended English learning cannot be explained without technologies (Grant et al., 2013), this study selects several possible factors to understand what learners learning performance is in metaverse-based blended English learning, what degree of learners' engagement in the virtual environments, What about the digital literacy of instructors and learner, and what the urgent issues are in the metaverse technologies. The contribution of this study is to know learners' benefits or any discomforts in metaverse-based blended English learning. Thus, this study might provide beneficial tips for researchers and educators to further study metaverse-based education.

## 2. The need for the study

### 2.1. Metaverse

Metaverse is an immersive three-dimensional (3D) virtual reality where multi-user as avatars interact and tend to create artificial societies in an online environment (Davis et al., 2009; Díaz et al., 2020). Avatars, software agents, are our alter egos (Kye et al., 2021). Virtual reality is a metaphor for the real world in which people engage daily activities and economic life through avatars (Kye et al., 2021). The metaverse denotes a world where social, economic, and cultural activities are conducted without barriers to collaboration (Jovanović and Milosavljević, 2022). It is different from the physical world. There is no agreed-upon definition of metaverses (Davis et al., 2009). Extensive studies have provided substantial evidence about the potential contribution to knowledge in several areas, such as unique technology capabilities and a deeper understanding of virtual collaboration.

### 2.2. Characteristics

The virtual reality metaverses have mainly three essential characteristics: interactivity, embodiment, and persistence (Castronova, 2001; Díaz et al., 2020; Kye et al., 2021; Almarzouqi et al., 2022; Makransky and Mayer, 2022). First, interactivity makes users interact and communicate with others in the metaverse through their avatars (Yen et al., 2013). Users impact the unrealistic objects and exert an influence on the opinions and behaviors of others; the appearance of avatars can reflect users' sense of presence and outcomes in virtual teams, which is reciprocal (Blascovich, 2002; Biocca et al., 2003; Díaz et al., 2020). Interactivity is the extent to which users can involve; it expands the possibility of individual or worldwide interaction (Díaz et al., 2020). Second, embodiment implies that avatars represent users in the metaverse (Blascovich, 2002). It symbolizes the participants' presence, including their suppositional environment and how avatars and the environment interact. The more realistic representation, the stronger the sense of presence and immersion, and the greater the users' engagement in the metaverse in contrast with face-to-face interactions (Blascovich, 2002). Third, persistence ensures the continuity of data (e.g., the position, conversations, property objects) to be saved. Moreover, data can be retrieved easily once the participants are reconnected or not connected to the virtual world (Abu-Salih, 2022). The metaverse platform continues to function and develop even after the users have departed from the virtual world (Almarzouqi et al., 2022).

### 2.3. Education-based metaverse for the blended English learning

The immersive technology of the metaverse brings about the new possibility of blended learning (Henderson et al., 2018; Kye et al., 2021). The infinite potential metaverse for educational applications may be an ideal replenishment for blended English learning (Henderson et al., 2012). The metaverse further breaks the limitations of online learning in access to knowledge (Thorne and Macgregor, 2018). The application of the education-based metaverse as the virtual classroom can be incorporated with face-to-face learning. A blended learning approach uses online learning facilitated the metaverse-based applications in a real classroom, creating an authentic English learning environment for learners (Thorne and Macgregor, 2018). An added value of using the metaverse in a blended learning environment is that it can develop learners' critical thinking and problem-solving, consistent with learning values and higher education teaching (Garrison and Kanuka, 2004).

Education-based metaverse refers to using metaverse-based platforms to improve learning activities (Wood and Gregory, 2018). The education-based metaverse creates an immersive environment that engages English learners in an authentic learning atmosphere (Reisoglu et al., 2017). In addition, the potential of the education-based metaverse can foster the development of skills needed by English learners (Reiners et al., 2018; Wood and Gregory, 2018). Such skills are the ability to collaborate with others, effective communication, and creative problem-solving (Dalgarno and Lee, 2010; Denson and Zhang, 2010). There are mainly five primary affordances of the education-based metaverse in English learning and teaching: the acquirement of English knowledge, the promotion of deep English learning activities, the enhancement of intrinsic motivation, sufficient provision of English learning opportunities, the improvement of self-efficacies, and the boost of rich and compelling collaborative English learning tasks from the virtual world (Dalgarno and Lee, 2010; Reisoglu et al., 2017).

### 2.4. Learner engagement in the education-based metaverse

The term engagement in the context of education-based metaverse means user engagement or learner engagement (O'Brien and Toms, 2008; Arnone et al., 2011). At the same time, the terms (student engagement, school engagement, or learner engagement) are utilized in teaching and learning (Reeve and Tseng, 2011; Romero, 2012; Fredricks et al., 2016). User engagement has become a significant factor in effective predict learner responses to a given learning task in the behavioral, emotional, and cognitive aspects (Reeve and Tseng, 2011). The education-based metaverse is considered a means to enhance user engagement for English learners at university (Jacka and Hill, 2018). In addition, the education-based metaverse offers a perfect, engaging visual environment, especially for young English learners (Jacka and Hill, 2018).

Based on the education-based metaverse, English users' engagement can be improved as they are offered immersive and interactive experiences through a virtual environment (Tlili et al., 2022a,b). English users can flexibly and study relatively quickly due to no physical restriction in the real world, which tend to elevate the engagement level of English learners (Jacka and Hill, 2018). All English learners are independent and can actively conduct action plans (Cho et al., 2022); thus, they are more engaged in achieving learning goals in education-based metaverse environments (Tlili et al., 2022a,b). The metaverse can provide technical support and autonomous tutoring systems, which help learners to establish correlations between virtual objects and experiments for English courses. So it provides new possibilities for engagement in learning the English language (Khan et al., 2022; Tlili et al., 2022a,b).

However, some literature put forth no direct relationship between the perception of engagement of English users t and virtual worlds based on the new education-based metaverse (Rowe et al., 2011; Jacka and Hill, 2018). Such engagement does not necessarily indicate language learning. There is also a concern that user engagement perhaps translates to off-task thoughts rather than English learning (Romero, 2012). Furthermore, time on distracting entertainment in game-based environments is wrongly reputed to be engagement but not English learning (Mayer and Johnson, 2010). Therefore, there is a clear need for further demonstration in blended English learning.

### 2.5. Learning outcomes in the immersive virtual worlds of the education-based metaverse

Immersion can be regarded as the subjective impression that a user submerses into a virtual environment, which is a loose psychological concept or an experience (Brown and Cairns, 2004; Dede, 2009; Reiners et al., 2018). In the education-based metaverse, successful temporal immersion in virtual teams is usually connected with higher performance (Davis et al., 2009; Thorne and Macgregor, 2018). The metaverse-based platforms support English learners with an overall immersive virtual learning environment which can be considered an educational simulation (Dede, 2009; Cheng and Ye, 2010). English learners can “meet” real people on the metaverse and communicate with them in their target language. In addition, Avatar representation can enhance English communication skills, whilst virtual worlds afford navigational and socialization tools which improve user presence (Schwienhorst, 2002). Immersion experience in 3D virtual worlds can support complex thinking, facilitate learning a second language, and positively influence better English learning outcomes (Grant et al., 2013).

Extensive descriptive research focused on English learning outcomes on the education-based metaverse (Kim et al., 2012; Grant et al., 2013). However, there is a lack of sufficient experimental research that may provide detailed data on ways the education-based metaverse could be utilized in English education. Furthermore, compared to limitations and inefficiencies in 2D learning environments, it is necessary to explore whether an immersive learning experience in the metaverse is advantageous in online learning (Mystakidis et al., 2021). Therefore, this research attempts to discover immersive atmospheres in 3D virtual worlds, which significantly aid English learning outcomes.

### 2.6. Digital literacy

Digital literacy is an important factor that may determine whether metaverse-based applications can be used to sustain education, which is related to the capabilities of learners in using the metaverses (Davis et al., 2009; Yu et al., 2022). To a certain degree, digital literacy influences instructors' and learners' intentions to participate in metaverse-based applications (Yuan et al., 2021). Digital literacy plays a vital role in Metaverse experience environments. Familiarity with metaverse-based applications leads to excitement and readiness for the sustainability of language learning. So instructors and learners need to increase their degree of digital literacy to use education-based metaverse flexibly (Abu-Salih, 2022).

Digital literacy on metaverse-based platforms can be considered a requirement for instructors and learners to skillfully use interaction techniques and acquire knowledge in virtual and physical environments (Gamelin et al., 2021). Digital literacy determines whether instructors and learners can access immersive learning environments and efficiently focus on English learning tasks in metaverse-based applications (Wang et al., 2015). Consequently, digital literacy exerts an important impact on learners' ability to carry out their daily adoption of metaverse technologies in a successful way (Hasse, 2017). Digital literacy can be improved by school training instructors and learners (Alvarez-Risco et al., 2022). In addition, an external effect such as institutional support ensures that instructors' and learners' digital literacy can be enhanced smoothly (Balakrishnan, 2017; Shao and Kwon, 2019). Moreover, previous studies reveal that instructors and learners should be a good grip on the entire scheme of information handling and audiovisual equipment (Diamantidis and Chatzoglou, 2018). In particular, English instructors and learners should be familiar with digital pedagogical tools to meet the needs of English courses in terms of digital literacy (Zhou, 2022). For English instructors and learners, it is an important ability to acquire knowledge and language skills in metaverse-based blended learning.

Based on related work, the metaverse-based technology has opened new possibilities for innovation in blended English learning (Díaz et al., 2020). In this sense, this study seeks to reproduce participation from the engagement perspective in an immersive virtual metaphor environment. In addition, engagement activities can be used to predict learners' learning outcomes. As learners become more engaged with technology-based learning and learning materials, their learning will increase (Chi and Wylie, 2014). This study suggests the potential of the metaverse as digital skills that support possible knowledge increase. However, the metaverse may raise daunting challenges on learners' and instructors' digital competence (Li and Yu, 2022). It is necessary to demonstrate a digital literacy level that motivates learners and instructors in their training. Meanwhile, digital literacy has become a significant driver in the choice of academic resources in blended language learning (Díaz et al., 2020). A learner may be pushed away due to the challenges during metaverse-based learning (Richter et al., 2022). With this in mind, we proposed alternative research questions.

#### 2.6.1. The aim of the study

Considering the metaverse as the new resource for the blended English learning, we propose the following research questions:

RQ1: Can the education-based metaverse promote English user engagement?
RQ2: Can the immersive virtual worlds of the education-based metaverse promote English learning outcomes?
RQ3: Is digital literacy an essential construct in metaverse-based blended English learning?
RQ4: What are the challenges on metaverse-based platforms?

## 3. Methods

**Figure 1:**
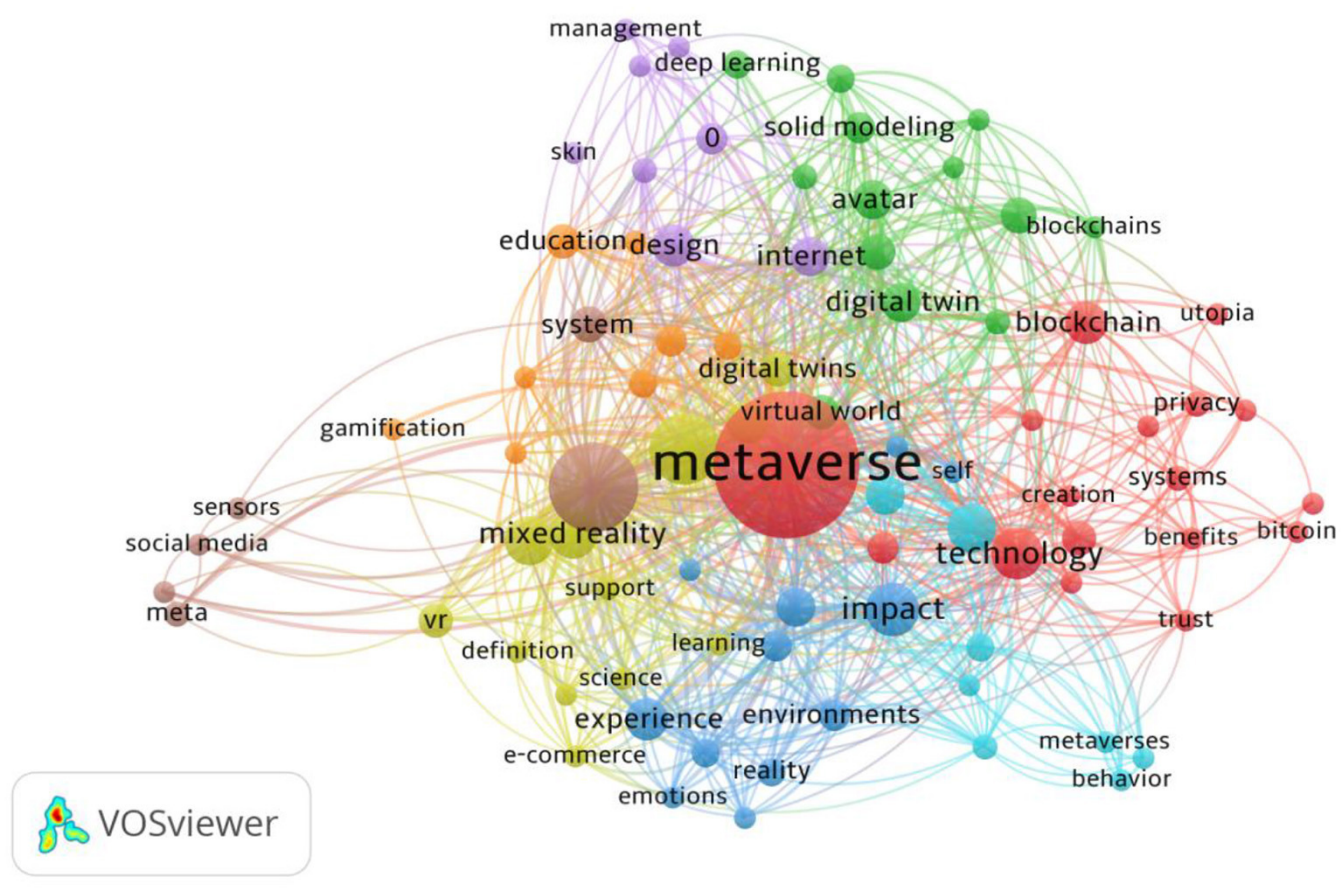
Clustering keywords related to the metaverse-based applications.

**Figure 2:**
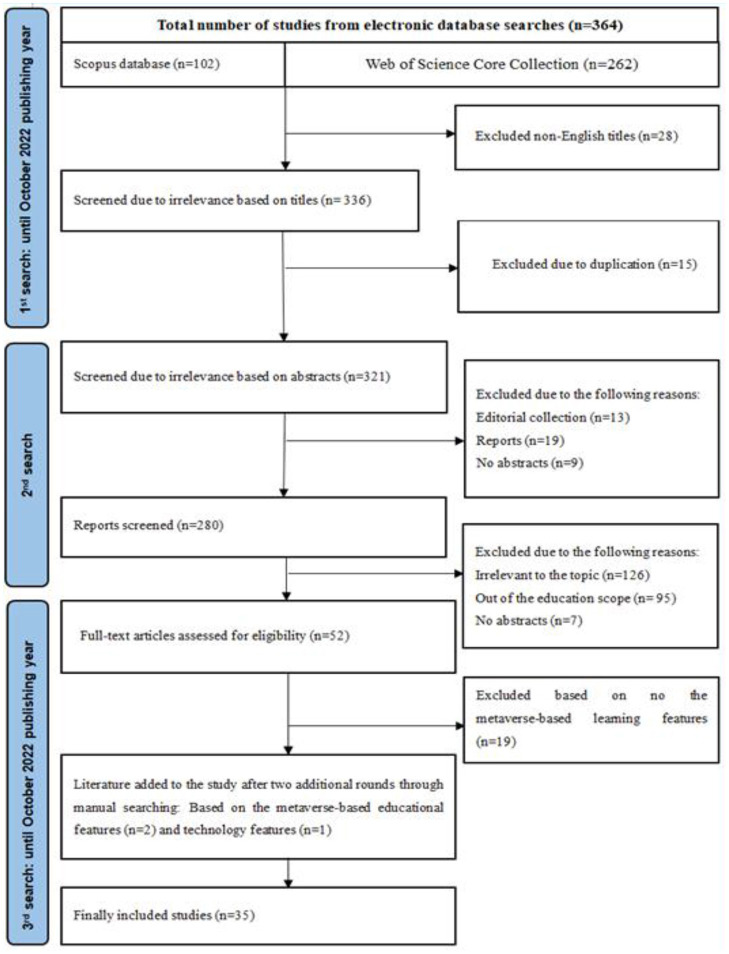
A flowchart of the literature inclusion and exclusion based on the PRISMA protocol.

This study adopted a systematic literature review on the current studies on the metaverse in English education. By a rapid evidence assessment, a four-step method was conducted to ensure that outcome reporting bias may be avoided because the interpretation of results tended to be subjective in manual censorship (He et al., 2017). So the study took a traditional bibliometric combined with a mixed-method systematic review and content analysis to identify the articles (Tlili et al., 2022a,b). The four steps were as follows:

- We planned the research themes and designed the research questions. The study got access to literature through widely searching for online databases. We obtained related literature through an extensive search for online databases. The search strings used in both Web of Science and Scopus databases were: metaverse (Topic) OR metaverse and education (Topic) OR metaverse and learn^*^(Topic) OR metaverse and English learn^*^(Topic) OR metaverse and English teach^*^(Topic). The data included academic articles published until October 2022.
- The study used clustering and mapping techniques in VOSviewer to verify research questions (van Eck and Waltman, 2011). VOSviewer is a software tool for constructing networks of scientific journals, keywords, or terms, which is mainly used for analyzing bibliometric networks through bibliographic coupling, co-authorship, co-occurrence, citation, or co-citation link (van Eck and Waltman, 2011). VOSviewer can primarily create visualized maps based on network data, which reflects research trends and hotspots among network and bibliographic database files (van Eck and Waltman, 2011). In this case, we selected the proposed research questions based on the scientific analysis of the full detailed output from VOSviewer. The study outputs the results (*N* = 262) in plain text and analyzes them *via* VOSviewer. Co-occurrence was selected as the analysis type; all keywords were chosen as the analysis unit. Full counting was used as the counting method. The minimum number of occurrences of a keyword was set at three. Of the 1,202 keywords, 84 met the threshold and were classified into six clusters (Figure 1). Cluster 1 included 17 items, e.g., benefits, bitcoin, blockchain, COVID-19, creation, cryptocurrency, customer experience, framework, governance, mental health, metaverse, privacy, systems, technology, trust, utopia. Cluster 2 included 13 items, e.g., artificial intelligence, avatar, avatars, big data, blockchains, deep learning, digital twin, games, information, machine learning, simulation, solid modeling, and virtual world. Cluster 3 included 12 items, e.g., acceptance, communication, emotions, environments, experience, immersion, impact, intention, model, reality, self, and telepresence. Cluster 4 included 12 items, e.g., augmented reality, definition, digital twins, e-commerce, extended reality, learning, mixed reality, science, support, technologies, virtual reality, and VR. Cluster 5 included 8 items, e.g., bibliometric, design, industry, internet, management, skin, and visualization. Cluster 6 included 8 items, e.g., behavior, collaboration, environment, metaverses, performance, second life, social presence, and virtual worlds. Cluster 7 included 8 items, e.g., education, gamification, mathematical models, online, satisfaction, students, technology acceptance, and virtual environments. Cluster 8 included 6 items, e.g., Facebook, meta, sensors, social media, system, and virtual reality.
- We calculated the total strength of the co-occurrence links with other key-words, and obtained a list of keywords with the top number of co-occurrence and link strength, among which the researchers found closely related keywords: metaverse (*N* = 126, link strength = 371), virtual reality (*N* = 47, link strength = 192), immersion (*N* = 5, link strength = 31), students (*N* = 5, link strength = 42), education (*N* = 7, link strength = 30), performance (*N* = 5, link strength = 28), learning (*N* = 3, link strength = 15). The study will evolve the topic based on these keywords and metaverse-based applications' challenges.
- To deal with this complex topic, we included and excluded the obtained results based on the Preferred Reporting Items for Systematic Review (PRISMA) (Moher et al., 2009) (Figure 2). PRISMA is adopted to allow researchers to estimate the applicability and credibility of the review findings (Page et al., 2021). It is designed for strictly evaluating and systematically reviewing research findings. The checklist items of PRISMA help facilitate and complete reporting of systematic reviews. In addition, the researchers used a critical appraisal tool (STARLITE) to score each selected article (Yu et al., 2021). STARLITE is already widely acknowledged as important in recording literature searches (Booth, 2006). Thus, reports of systematic reviews could be trustworthy. Complete literature should include a title, abstract, introduction, literature review, methods, results, discussion, and conclusion. The researchers worked out guidelines for systematic reviews. Two researchers collaborated to review the literature with high inter-rater reliability (*k* = 0.91). If they cannot agree on any decision, a third international researcher will be invited to determine the selection. A study was included if it (1) focused on metaverse-based education; (2) provided enough information for the study, or (3) arrived at clear conclusions. A study was excluded if it: (1) discussed metaverse in general and not in education; (2) was not in English; and (3) was not accessible online. As a result, 262 studies in the Web of Science database and 15 in the Scopus database were identified.

## 4. Results

### 4.1. The metaverse-based blended English learning

The metaverse-based blended English learning will improve the depth and breadth of English learning activities and speed the development of teaching-learning relationships (Makransky and Mayer, 2022). It is likely to fundamental reshape English education (Mughal et al., 2022). The issue of how to innovate blended learning has appeared significant during the post-pandemic era (Yu, 2016; Yu et al., 2022). At the same time, the education-based metaverse develops and fulfills all educators' imaginations (Reisoglu et al., 2017). Learners can communicate via avatars by applying audio- or text-based tools. Immersive 3D language learning environments enhance learners' engagement and better learning performance (Ryu et al., 2022). Virtual interaction with the content they favor stimulates learners' intuition and willingness. Learners can share information and receive multifaceted feedback. Learners can improve their intercultural communication level and problem-solved skills through avatars, which can bring learners satisfaction improvement and English academic success (Kye et al., 2021). Moreover, instructors can offer learners high-quality education, and learners' needs can be fully fulfilled in virtual classroom environments (Díaz et al., 2020). Thus, the traditional teaching-learning relationships can be significantly improved.

However, researchers have concerns that the mixture of the metaverse in education is a hoax. Because today's technology lacks trust and knowledge, the metaverse should not be applied to education on a large scale (Mughal et al., 2022; Qin, 2022; Yue, 2022). Moreover, the value of the education-based metaverse lacks sufficient confirmation, affecting its application to education (Makransky and Mayer, 2022). Still, there is a lack of systematic review on the educational value of using the metaverse (Makransky and Mayer, 2022).

### 4.2. RQ1: Can the education-based metaverse promote English user engagement?

The online 3-D virtual metaverse can affect learners' levels of engagement (Jacka and Hill, 2018). It has been proven through several studies that the metaverse provides users engaging in myriad learning activities, such as language classes (see, e.g., Diehl and Prins, 2008; Thorne and Macgregor, 2018; Lee et al., 2022). Multiple modes of interaction in physical and virtual worlds make the metaverse an ideal tool for examining user engagement (Diehl and Prins, 2008). Even though some learners may feel physical isolation, they can actively create avatars and participate in social or educational activities with other avatars (Lee et al., 2022). Although literature concerns that English learner engagement may be related to game-based environments in the metaverse rather than deep learning, some empirical studies have proved that a user learning environment with the gameplay design is critical to ensure engagement that leads to language learning and academic achievements (Jacka and Hill, 2018). In the context of English education in China, user engagement in learning English as a foreign language can improve *via* 3D avatar interactions in virtual space (Henderson et al., 2012).

The metaverse as an intercultural 3D online environment affords English learners to engage in purposeful virtual online activities through avatar-based perceptual learning (Zheng et al., 2009a). Furthermore, high levels of user engagement usually mean more freedom and effective manipulation in virtual environments (Schwienhorst, 2002). English learners can study performance, tourism, and exhibitions in virtual English spaces based on a high degree of self-engagement, which can directly influence or indirectly affect learning outcomes (Kye et al., 2021). Interestingly, the learner engagement degree may lower at the beginning period of utilizing the metaverse technology. Some learners feel thwarted about English learning in an immersive virtual environment, but they can adapt to an appealing, inclusive online platform (Thorne and Macgregor, 2018).

### 4.3. RQ2: Can the immersive virtual worlds in the education-based metaverse promote English learning outcomes?

Virtual worlds in the metaverse empower learners' learning outcomes and increase the overall personal learning experience (Mughal et al., 2022; Niaz et al., 2022). In the context of English education, the metaverse enables learners to construct English knowledge and discourses with peers in virtual worlds, which makes it easy for learners to produce English academic achievement (Henderson et al., 2018). In addition, with virtual reality, “real world” online chatting, learners gain a powerful channel to learn English (Almarzouqi et al., 2022). Thus, metaverse-based blended English learning can be an appropriate way to accomplish English learning tasks (Wood and Gregory, 2018). In particular, the education-based metaverse creates an immersive learning environment similar to a real classroom (Fardinpour et al., 2018). English learners can apply their knowledge to solving complicated problems that are the same as those in a physical learning environment (Grant et al., 2013). Especially in English vocabulary and grammar learning, virtual environments can provide learners with synchronous interaction and competency-based training, which is quite different from other online language learning tools (Reisoglu et al., 2017).

The metaverse technologies make English education more attractive because they support immersive learning (Chun and Plass, 2000). The current metaverse has shown unique advantages in promising results of English learning achieved by learners' immersive learning and their avatars (virtual persons). It has to be interestingly mentioned that immersion in metaverse-based technologies involves objective features of superb instructional styles that can bring learners an amazing, immersive experience (Levy, 2009). Immersion is not a psychological sense of being there, but it can objectively assess the degree of a system's virtual environment (Makransky and Mayer, 2022). Immersive virtual environments in the metaverse are projected to boost learners' learning to be more engaging and lifelike in unfamiliar circumstances (Dahan et al., 2022; Mughal et al., 2022).

### 4.4. RQ3: Is digital literacy an essential construct in metaverse-based blended English learning?

Overall, instructors and students lack digital literacy in using educational metaverse (Zheng et al., 2009a; Díaz et al., 2020; Kye et al., 2021; Lim et al., 2022; Yang et al., 2022). The technical skills of interacting in the metaverse significant predict levels of digital literacy (Díaz et al., 2020). Digital literacy is not merely a simple competence to use state-of-the-art technology to improve learning but a process that can continuously change and adapt to improve learning and teaching quality (Reiners et al., 2018). Literature has pointed out that high-tech equipment, immersive virtual environments, and a practical text-based course design in an education-based metaverse are essential for teacher digital literacy (Almarzouqi et al., 2022). On the other hand, learners may not be familiar with the metaverse and its use for learning, and a purposeful sampling technique should be introduced to learners (Cho et al., 2022).

Cultivating digital literacy must be improved to support instructors and learners to access metaverse technologies (Tlili et al., 2022a,b). The ability to use the digital system does affect the willingness and readiness of English learners to organize language learning activities in the metaverse era (gugebeiyong7-1). Furthermore, a lack of digital literacy in using the metaverse will cause certain technology anxiety among learners, especially “technology-naïve” or underachieved learners (Li et al., 2022). The metaverse-based technologies require the improvement of instructors' and learners' digital literacy (Grant et al., 2013). There is still a skill gap between using the new platforms and the competence of instructors and the learners' digital literacy (Levy, 2009). In addition, the degree of digital literacy is an important factor in sustaining online English education (Tlili et al., 2022a,b). For instance, if English instructors can quickly apply different technological means to use digital resources, English learners can be more intuitive to adopting online learning (Díaz et al., 2020).

### 4.5. RQ4: What are the challenges on metaverse-based platforms?

The high degree of freedom of the metaverse has appeared to be some challenges in applying language education (Yang, 1998; Reiners et al., 2018; Thorne and Macgregor, 2018; Dayoub, 2022; Zhou, 2022). Challenges may include gamification, social distancing, and internet availability.

#### 4.5.1. Gamification

The metaverse provides game design to extend learner learning experiences, but the game sessions may weaken learner engagement and lead to terrible learning outcomes (Zheng et al., 2009b). The metaverse is considered the only online game, a significant risk to young learners. The metaverse tools have unique characteristics, such as 3D interactive simulation and real-world physics, so the platform's learning process is similar to gamification (Dalgarno and Lee, 2010). In addition, a gamified interaction may lack sufficient instructor supervision. It is difficult for instructors to effectively supervise learners' immersive learning activities with a virtual presence (Jovanović and Milosavljević, 2022). Fear of high self-regulation and self-control, learners, tend to obtain poor learning outcomes.

#### 4.5.2. Social distancing

The metaverse, which provides high immersion through visualization, may cause users to feel socially distant as learners, as it attracts users with the same hobbies and interests to interact and communicate in virtual environments (Fola-Adebayo, 2019). The metaverse may cause a risk of learner isolation, although it creates learner autonomy (Alvarez-Risco et al., 2022). It supports virtual social connection even in the real world, which may lead to social distancing or social alienation (Fola-Adebayo, 2019). Therefore, the metaverse may weaken social interactions (Zhou, 2022). In the field of English education, users may transmit the wrong information, “me I want to show” instead of “me as I am,” so that the instructor and administrators cannot predict the actual intercultural communication competencies and language skills in the virtual worlds (Puncreobutr et al., 2022). This case is more dangerous than learners of existing online games (Kye et al., 2021). Furthermore, underachieved learners with low engagement or weak self-control may feel hard to adapt to the metaverse technologies. Thus, they tend to feel social alienation (Kye et al., 2021).

#### 4.5.3. Deficient support

The metaverse technologies have foregrounded deficient education support (Puncreobutr et al., 2022). The metaverse-based education heavily depends on the economic status of universities (Zhou, 2022). Policymakers and administrators may estimate the improvement of the education reform and learner educational benefits on all files (Poddubnayaa et al., 2020). Technical input is another challenge facing metaverse-based education. The internet is the main factor for online learning effectiveness (Dayoub, 2022). Due to the inconvenient Internet connection, the metaverse platforms are still totally or partially out of service (Lim et al., 2022). Consequently, instructors and learners may feel hesitant to access or interact in the metaverse-based learning process, much less within the specified time of the course (Yang, 1998).

## 5. Discussion

The study attempts to integrate the evidence to generate a model that links the education-based metaverse. The metaverse platforms in which learners' academic success can be significantly enhanced due to a high degree of learner engagement in immersive virtual environments. In addition, the virtual learning experience is restricted by the degree of digital literacy at the same time. To improve instructors' and learners' digital literacy levels, necessary support is indispensable by educational institutions and developers and designers of the metaverse platforms.

### 5.1. A new direction of the blended learning

The metaverse-based blended learning plays an important role in linking the virtual worlds and the context of real face-to-face classroom learning (Díaz et al., 2020). It can effectively improve learners' academic performance by providing the immersion principle in 360-degree panoramic images (Almarzouqi et al., 2022). The metaverse technologies enable an individualized and encompassing English learning experience that ranges from a real classroom English education context to a virtual environment (Dayoub, 2022). The unique two-way interaction supported by the metaverse technologies lays the foundation for sustainable English learning (Fola-Adebayo, 2019). The metaverse-based blended learning can significantly improve learners' engagement and their learning outcomes.

Due to the rapid development of metaverse technologies, the value of integrating immersive virtual environments into blended English learning has brought awareness to educators and researchers (Fola-Adebayo, 2019). Undoubtedly, it is still in its infancy using metaverse-based blended learning (Yu, 2016). Concerns with the economy, culture, politics and even personal information security caused by the metaverse technology can be solved properly (Zhou, 2022). Furthermore, developers and designers of the metaverse platforms could design easier and more convenient tools which can directly or indirectly stimulate language instructors' and learners' readiness in the teaching-learning process (Puncreobutr et al., 2022).

### 5.2. Perceived learner engagement

The observed degree of learner engagement based on the education-based metaverse can be comprehensively improved (Thorne and Macgregor, 2018). The education-based metaverse can improve English learner engagement by providing 3D recreation and language learning opportunities (Henderson et al., 2018). However, some learners may hesitate to the metaverse technologies and not be ready to engage at the onset of learning (Thorne and Macgregor, 2018). Sustainable engagement calls for more interaction with the instructors and peers. Especially for mature students in English learning, instructor support is primary for them to fulfill English learning activities in the metaverse environment (Thorne and Macgregor, 2018). Familiarity with the platform and accomplishment of the learning tasks with instructor assistance are indispensable parts of learner engagement.

Moreover, it is meritorious to facilitate the lower degree of English learners' engagement in online virtual learning ease (Jacka and Hill, 2018). A blended learning environment utilizing the metaverse may concern English learners' ability to engage, or English learning in a virtual world causes frustration and annoyance, which trigger learner distraction and a lower degree of engagement (Thorne and Macgregor, 2018). It is beneficial to build up work teams or collaborative learning to passionate them to engage in learning in a wider community of virtual space (Lee et al., 2022). The instructors can also provide one-to-one guidance to help them ease learning anxiety in a virtual world (Abu-Salih, 2022). However, low-level resistance to engaging with the metaverse may still exist, as revealed in learners' English pedagogy questions or the metaverse platforms.

### 5.3. Learning outcomes improvement

The immersive learning experience positively affects learners' emotions and learning effectiveness which influence the engagement of English learning, activate learners' autonomy, and determine learning outcomes (Makransky and Mayer, 2022). Metaverse technology generates immersion that differs from psychological immersion (Makransky and Mayer, 2022). Technology-based immersion relates to the depth of users' involvement with academic activities in a gameplay model (Cho et al., 2022). Well-designed games with a given task can foster learners' learning performance with immersive virtual environments (Gamelin et al., 2021). However, it is impossible to provide relative complicate games to support language learners' multi-dimensional learning activities in the real world (Reiners et al., 2018). Gameful experiences can promote users' intrinsic and extrinsic motivations to achieve academic success in the virtual world (Reiners et al., 2018). Appropriate gamification design in the metaverse technology can stimulate users' willingness to get higher grades in blended English learning (Cho et al., 2022).

Furthermore, metaverse-based immersive language learning can cultivate learners' intercultural awareness and critical thinking in authentic virtual worlds. Consciousness and thinking can help language learners become more likely to be closer to the target language community (Wood and Gregory, 2018). The metaverse's main feature is that it solves the language learning scenario's main constraints through authentic virtual environments (Almarzouqi et al., 2022). The virtual environments, as the mediation of tacit knowledge, provoke deeper-level thinking processes, which is crucial to the effectiveness of English language learners. Some successful language learners are adept at innovative learning resources of virtual worlds to achieve better learning outcomes.

### 5.4. Digital literacy enhancement

Digital literacy is important for accomplishing learning tasks in the metaverse platforms. Learners and instructors are not viewers in the virtual world of the metaverse (Puncreobutr et al., 2022). It would be beneficial to elevate their degree of digital competitiveness in the metaverse (Yang et al., 2022). For instance, learners may accomplish the primary training tasks: basic knowledge of the metaverse technology, major features of the metaverse devices, and the use of the metaverse applications (Poddubnayaa et al., 2020). Instructors' digital literacy ability requires fundamental knowledge of metaverse technologies (Poddubnayaa et al., 2020). Thus, it is necessary to establish a motivation system for mastering the knowledge and skills of the metaverse because it has been noted that the technology and resources to improve the digital literacy of English learners and instructors are insufficient, especially in teacher education schools (Yue, 2022).

Moreover, some aspects of digital literacy need to be polished based on digital pedagogical discipline models (Qin, 2022). For instance, the technology training of English instructors needs follows the type of metaverse tools which can consolidate English teaching and learning. In addition, instructors can be familiar with instructional and technological interactions (e.g., exchanges between students, managing the software, handling computer-based prompts, etc.) (Tlili et al., 2022a,b). Instructors also need to make sense of the entire system of metaverse-based applications (Dahan et al., 2022). Furthermore, a high degree of digital literacy can significantly promote the development of the compulsive competence use of communication and information technologies in higher English education (Diehl and Prins, 2008). Therefore, digital literacy on metaverse-based applications requires expanding English instructors' knowledge of specific digital tools and management, and instructors' interaction with learners can be quick in metaverse-based platforms (Levy, 2009). Therefore, the enhancement of digital literacy should adapt English study plans; training activities might be dynamic to follow related curricular criteria (Díaz et al., 2020). It is necessary to improve instructors' and learners' digital literacy according to context-appropriated frameworks for language learning (Lim et al., 2022). The perceptive satisfaction degree of digital literacy can be gauged, ranging from instructor to learner based on their attitude toward English courses (Niaz et al., 2022).

### 5.5. Embracing challenges

The metaverse-based smart education provides us with more opportunities as well as challenges. Based on the high degree of freedom, instructors should be cautious that learners misuse educational metaverse platforms (Zhou, 2022). Gamification in enhancing academic achievement has two faces: it provides shared 3D entertainment and learning opportunities (Reiners et al., 2018; Zhou, 2022). It is necessary for instructors to carefully evaluate learners' learning activity patterns, level of immersion in the metaverse, and their behavioral intentions to use the metaverse (Makransky and Mayer, 2022). An innovative education strategy should cover each teaching link to help learners achieve learning goals (Almarzouqi et al., 2022). To optimize the usefulness of metaverse-based language education, instructors should manage various resources to fulfill specific language learning tasks under the learning model by doing in the metaverse applications (Díaz et al., 2020). In addition, instructors and managers can act as facilitators and guides to assist learners in their training (Reiners et al., 2018).

The metaverse-enhanced learning can be monitored to avoid learners feeling socially distant (Davis et al., 2009). Educators and administrators can use more effective English learning materials and more exact virtual environments that can fully foster learning activities (Schwienhorst, 2002). They can gradually improve the function to increase learners' perceptions of being in the “real world” and belonging to a learning community. The metaverse platform developers may pay more attention to designing 3D virtual learning worlds that emphasize emotional achievements, learning support, and the desired learning outcomes (Dalgarno and Lee, 2010; Reisoglu et al., 2017). They can design online applications to enhance supervision over learning behaviors and provide reminders when learners behave improperly (Alvarez-Risco et al., 2022).

Possible solutions can be taken into account to financially and technologically support metaverse-based education (Yang, 1998; Zhou, 2022). Policymakers and administrators could conform to the educational trend. Otherwise, traditional education models will be hard to support remote education, especially during the emergent period (Dahan et al., 2022). We have amazed to see that leading universities in China and worldwide have developed and implemented their own 3D virtual campus environments in instructional processes (Reisoglu et al., 2017). The metaverse developers should improve the compatibility of different hardware and software to reduce instructors' and learners' stress and anxiety (Ng et al., 2022). The developers can design offline sections to enlarge opportunities to access the metaverse platforms. Digital infrastructures could solve technical issues and establish a specialized department to manage and supervise the metaverse platforms (Alvarez-Risco et al., 2022; Lee and Hwang, 2022).

## 6. Conclusions

### 6.1. Major findings

This study aims to investigate the education-based metaverse to sustain the current blended English learning during the post-pandemic era by examining some significant factors. The main factors are learner engagement, learning outcomes, and digital literacy. Meanwhile, this study addresses potential challenges that may hinder sustaining metaverse-based blended English learning.

Once the metaverse mixes English education, it can reshape the prior blended English learning. The metaverse can provide affluent, direct, and indirect learning opportunities, greatly enhancing English learners' learning readiness and intuition. The education-based metaverse can positively promote English user engagement. The immersive learning experience in the education-based metaverse can positively promote English learning outcomes. Digital literacy on metaverse-based technologies is an essential construct in metaverse-based blended English learning. The features of high freedom of the metaverse have appeared as challenges in the application of language education. Challenges may include gamification, social distancing, and internet availability. This study has provided some suggestions according to the previous literature.

A quality learning environment is meaningful learners do better at creative and critical thinking skills (Garrison and Kanuka, 2004). The most significant aspect of this study has been the need to explore the opportunity to enhance the effectiveness and efficiency of teaching and learning, which may bring successful responses to learners' needs, such as wellbeing and positive emotion. The core values of learner positive emotion can produce a psychological state favorable to learning (Li et al., 2021). Furthermore, with advances in information and communication technologies, blended learning can be well-updated through technology-meditated education, such as the metaverse and other online learning tools. This study aims to reveal that the metaverse-based technology implements and sustains effective blended learning environments, which may enhance learner spirits and create positive momentum for language learning.

There are four types of the metaverse which have developed independently: (1) Augmented reality: a smart environment can be built to augment the real physical world based on location-based technology (Bacca et al., 2015). A representative educational case is that language learners can effectively obtain learning materials that cannot be presented in texts *via* smartphones or vehicle HUDs (head-up display) (Kye et al., 2021). When language learners require continuous experience or practice, augmented reality technology allows learners to link abstract visuals to concrete objects and solve English reading, writing, or speaking problems (Han and Lim, 2020). (2) Lifelogging: a world of lifelogging is an augmentation of the inner world that can be recorded and analyzed through augmented technology (Kye et al., 2021). For example, learners' daily learning activities and thoughts can be shared through social media, such as Facebook and Apple Watch (Kye et al., 2021). A typical example of education is that learners' learning achievements can be analyzed. For instructors, it provides them valuable feedback, which leads to reinforcement and an appropriate teaching direction (Dwivedi et al., 2022). (3) Mirror world: a simulation or reflection of the real world (Bacca et al., 2015). All activities in the mirror world can be done based on mobile applications (Dwivedi et al., 2022). A representative of a mirror world metaverse used in education is digital laboratories or real-time online classes *via* online video conferencing tools (such as Zoom and Tencent Conference) (Kye et al., 2021). Learners can overcome the physical or special limitations of learning in the mirror world metaverse (Kye et al., 2021). English learners can realize non-face-to-face remote learning, which presents great educational potential (Dwivedi et al., 2022). (4) Virtual reality: a combination of a virtual world and real-world built with digital data is, to some degree, the most complete of virtual reality (Hollensen et al., 2022). This virtual reality metaverse dramatically enlarged the scope of educational spaces based on a complex of virtual reality technologies (such as avatars, 3D graphics, and instant communication tools) (Kye et al., 2021). A representative example of metaverse-based education is that learning can be performed *via* instant communication tools (such as Second Life, Minecraft, Roblox, and Zepeto) (Hollensen et al., 2022). English learners can simultaneously access and perform meaningful tasks in immersive virtual environments (Kye et al., 2021). The metaverse provides customized learning levels (Hollensen et al., 2022). Furthermore, it breaks the boundaries between the abovementioned types of metaverse (Kye et al., 2021).

This study provides theoretical implications and practical guidelines for educators and researchers to enhance the metaverse-based blended English learning construction. This study shows that higher-immersion environments create higher levels of presence and better learning outcomes. Based on the characteristics of the metaverse, the researchers support that the metaverse is an evolved concept. More importantly, metaverse-based education performs important missions in blended education contexts. The metaverse platforms provide not merely online games but self-directed learning experiences. On the other hand, the metaverse makes it possible to expand the scope of English education. It is predicted to improve learners' academic performance and significantly expand their freedom to explore questions. However, appropriate regulations and measures are necessary for both instructors and learners to adopt metaverse-assisted methods for educational purposes.

### 6.2. Limitations

There are several limitations to this study. First, it should be acknowledged that studies cited in this review may be insufficient, owing to the databases and keywords used in this systematic review. Second, the findings of this study are limited by the author's resource availability. Third, this study only reviews English academic articles about the metaverse in education. Some interesting and meaningful findings might be neglected, which might lead to the deviation of the research findings in this study. Fourth, due to the immaturity of the metaverse and lack of sufficient published studies identified from the Web of Science and Scopus databases, this study cannot capture the whole picture of the research.

### 6.3. Future research directions

In future research, we will keep updating and polishing the metaverse-based blended English learning research. Due to the immaturity of metaverse-based blended English learning, this study cannot provide the overall research findings. In addition, we may add more practical features, such as self-efficacy, the degree of satisfaction, technology anxiety, etc. With advances in metaverse technology, we may enrich the systematic review by building a theoretical model on metaverse-based blended English learning. Meanwhile, instructional tools and advanced evaluative measures will be used.

Another direction of future research on metaverse-based blended English learning may be to reveal the metaverse-based applications and their effects on learners' motivation through an empirical study. The findings of this study show that metaverse technologies can enhance the efficiency of language learning and improve learners' learning outcomes; at the same time, the mixture of the metaverse has brought some challenges, such as a lower level of instructor and learner digital literacy. In the future, it is necessary to empirically investigate the advantages and disadvantages of the metaverse to sustain blended English learning. We may empirical studies to analyze and calculate the effect of the metaverse on blended English learning in higher education.

## Author contributions

Both authors listed have made a substantial, direct, and intellectual contribution to the work and approved it for publication.
